# One Brain, One Vision

**DOI:** 10.1371/journal.pbio.0020414

**Published:** 2004-10-26

Not all devices that measure the same property do it in the same way—a clock might use a spring system or it might be digitally synchronized to a transmitted signal. Although both have the same goal of reporting accurate time, each is subject to different errors. Sometimes even the same device uses different systems to measure the same property. A relatively simple device like a camera will use one sensor system to capture light intensity for an image and a second sensor to capture light intensity for making automatic adjustments of aperture and flash. It does not seem outlandish, therefore, that the brain might also have developed multiple sensory systems to achieve different goals. Indeed, an influential hypothesis has argued that people use two separate visual processing systems in much the same way as a camera—one for creating our perception of the world and another for guiding our actions within it.

One line of evidence supporting this dual hypothesis comes from an illusion known as Roelofs effect. Usually, people are pretty good at judging the location of even a small object. But if the small object is surrounded by a large frame and the frame itself is not centered in front of the person who is judging it, the viewer will perceive the object as shifted in a direction opposite that of the frame. This may not in itself be surprising, but the same person who perceives an offset of the object where none exists is nonetheless able to grasp it without difficulties.

**Figure 1:**
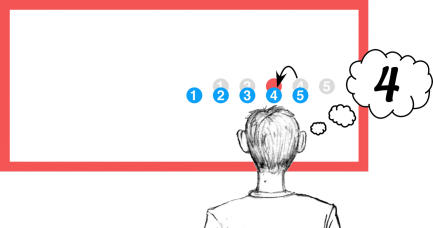


In this issue of *PLoS Biology*, Paul Dassonville and his colleagues reexamine the seeming dissociation of visual analysis for perception and action, and call it into question. Through a careful quantitative analysis of the conditions under which the Roelofs effect occurs, they find that it traces not to an illusory perception of the object location but to an illusory perception of self. The large frame, presented under experimental conditions in which subjects sit in darkness without access to a normal rich sensory environment, actually causes people to incorrectly perceive their own centers as rotated towards the frame and therefore to conclude that the small object is offset with respect to themselves. This may seem like a subtle distinction, and yet, since it is the observer's frame of reference that is altered, that same distorted frame of reference will be used to guide movement. Thus, the error in movement planning should cancel the error in perception, and people should have no trouble reaching for the object despite their misperception, which is indeed what is observed.

Others have questioned the hypothesis that two separable neural systems process the visual world for perception and action, but this study removes one of the strongest pieces of evidence in its favor with a precise alternative explanation. No two brains may see the world identically, but the authors suggest that it may be time to concede that a single brain, at least, has the same world view.

